# Supplementary material for: The membrane associated accessory protein is an adeno-associated viral egress factor
Source: Nat Commun. 2021 Oct 29;12:6239. doi: 10.1038/s41467-021-26485-4 (PMC8556243; doi:10.1038/s41467-021-26485-4)
Supplement: Supplementary file 1 — Supplementary Information [file 41467_2021_26485_MOESM1_ESM.pdf]

Supplementary Information

The membrane associated accessory protein is an adeno-associated viral egress factor

Zachary C. Elmore[1], L. Patrick Havlik[1], Daniel K. Oh[1], Leif Anderson[2], George Daaboul[2], Garth W. Devlin[1], Heather A. Vincent[1], Aravind Asokan[1,3,4,5*]

[1]Department of Surgery, [2]Nanoview Biosciences, Boston, Massachusetts, USA, [3]Department of Molecular Genetics & Microbiology, Duke University School of Medicine, Durham, NC, USA, [4]Department of Biomedical Engineering, [5]Duke Regeneration Center, Duke University, Durham, NC, USA,

*Corresponding Author: Aravind Asokan, Ph.D.

5148, MSRB3,

3 Genome Court,

Durham NC 27710, USA

Email: aravind.asokan@duke.edu

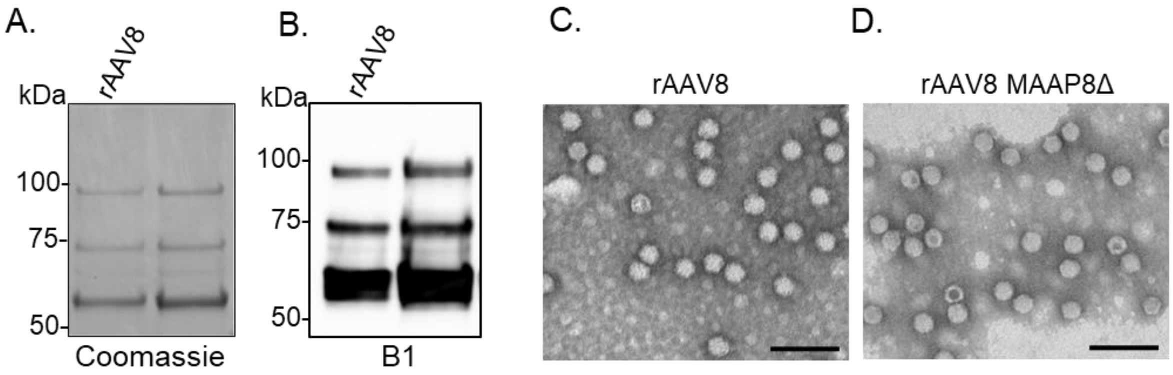

Supplementary Figure 1. MAAP ablation does not affect AAV capsid protein composition and morphology. (A) Recombinant AAV8 and AAV8 MAAPΔ virus was purified from the media of HEK293 producing cells. AAV8 and AAV8 MAAPΔ viral capsids were analyzed by SDS-PAGE under reducing conditions and stained with coomassie **(A)** or probed with a capsid (B1) specific antibody **(B)**. Gel and immunoblot are representative images from 3 independent experiments. TEM images of rAAV8 **(C)** and rAAV8 MAAPΔ **(D)** viral capsids. TEM micrographs are representative images from 2 independent experiments. Scale bars, 100 nm.

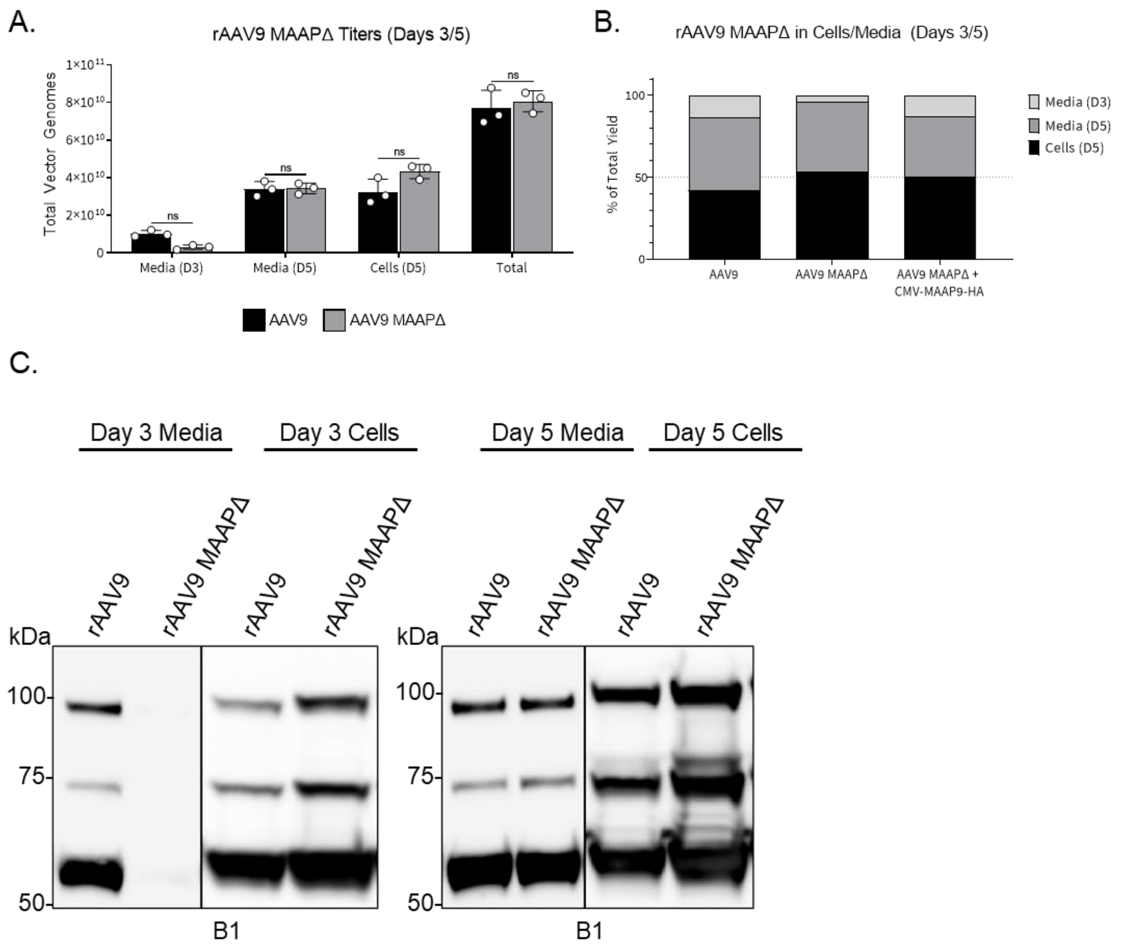

Supplementary Figure 2. Ablation of MAAP expression differentially impacts recombinant AAV9 secretion. (A) AAV9 ssCBA-Luc vectors produced with WT Cap or MAAPΔ Cap. Total vector genomes collected from the cells and media and the proportion of virus found in each media harvest or associated with the cells **(B)** are shown. Each bar is a representation of three experiments that are biological replicates. Data are presented as mean values +/- SD. Significance was determined by two-way ANOVA, with Sidak's post-test.. $^{n.s.}p \geq 0.05$. **(C)** Recombinant AAV9 and AAV9 MAAPΔ viruses were analyzed from the media and pellet of HEK293 producing cells at days 3 and 5 post transfection. Capsid proteins were analyzed by SDS-PAGE under reducing conditions and probed with a capsid (B1) specific antibody. Immunoblots are representative images of 2 independent experiments.

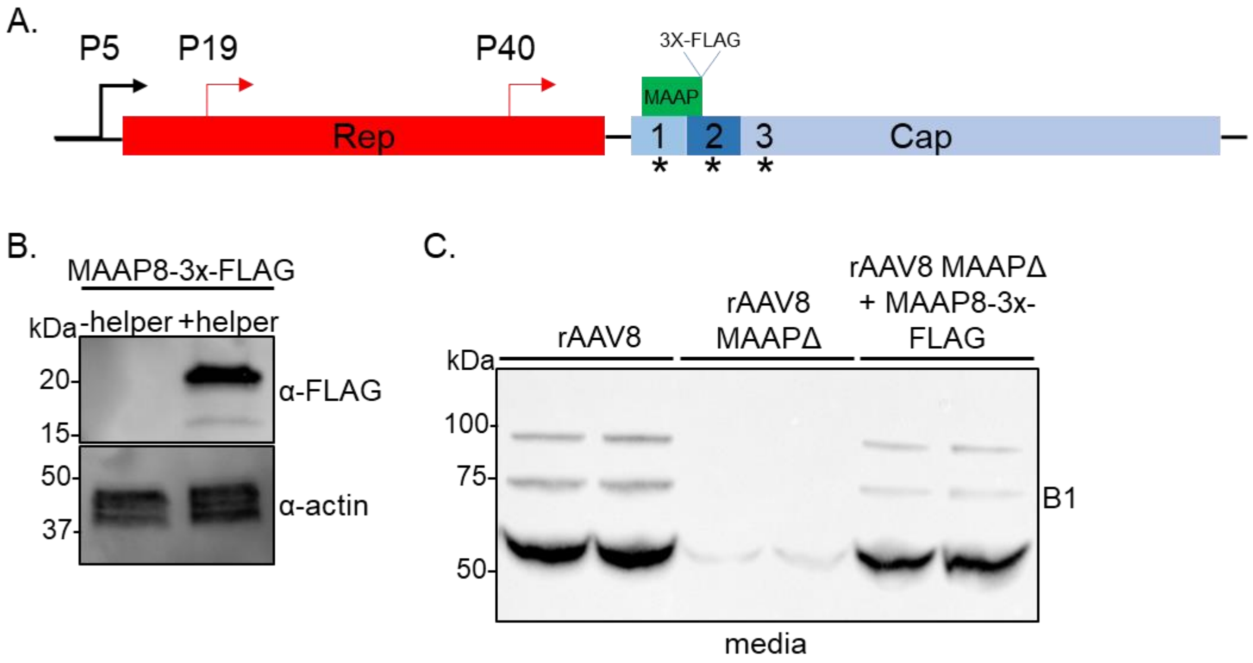

Supplementary Figure 3. MAAP8-3X-FLAG rescues MAAP8Δ function.

(A) Schematic of recombinant AAV8 VP/AAP-null MAAP8-3X-FLAG (MAAP8-3X-FLAG) plasmid used to replicate endogenous levels of MAAP expression. **(B)** HEK293 cells were transfected with MAAP8-3X-FLAG along with pXX680 (Adenoviral helper) plasmids and harvested 72 hours post transfection. Whole cell lysate was analyzed by SDS-PAGE under reducing conditions and probed with FLAG (α-FLAG) and actin (α-actin) specific antibodies. Immunoblot is a representative image of 2 independent experiments. **(C)** Recombinant AAV8 and AAV8 MAAPΔ viruses complemented with MAAP8-3X-FLAG were analyzed from the media of HEK293 producing cells at day 3 post transfection. Capsid proteins were analyzed by SDS-PAGE under reducing conditions and probed with a capsid (B1) specific antibody. Immunoblot is a representative image of 2 independent experiments.

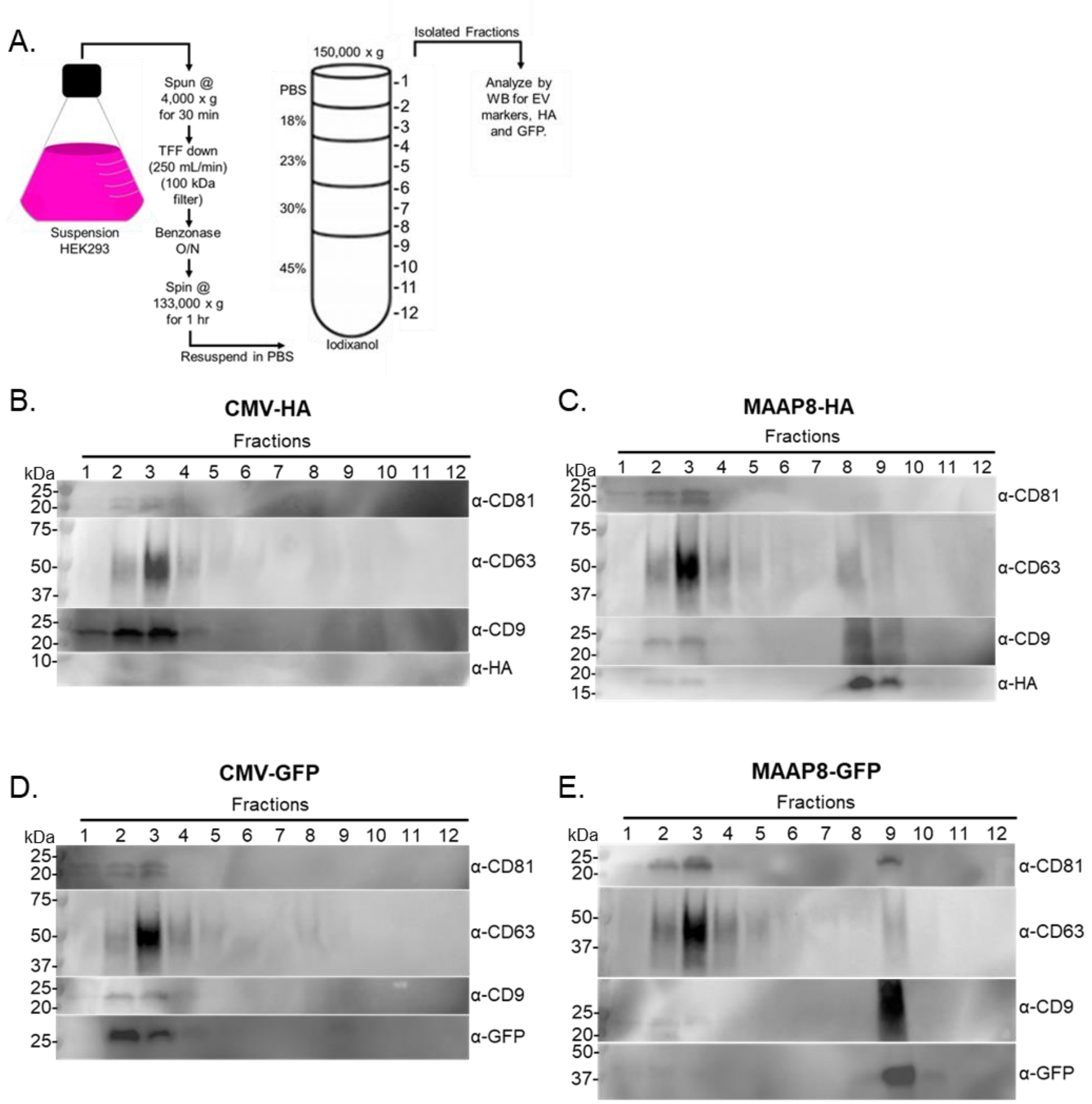

Supplementary Figure 4. MAAP colocalizes with distinct EV fractions.

(A) Schematic of EV isolation by iodixanol density gradient from HEK293 suspension culture. Immunoblots of iodixanol fractions from suspension cells expressing **(B)** CMV-HA, **(C)** CMV-MAAP8-HA, **(D)** CMV-GFP, **(E)** CMV-MAAP8-GFP. EVs and MAAP were analyzed from the media of HEK293 producing cells at day 3 post transfection. MAAP proteins (HA, GFP) were analyzed by SDS-PAGE under reducing conditions while EV markers (CD81, CD63, CD9) were analyzed by SDS-PAGE under non-reducing conditions (n=2).

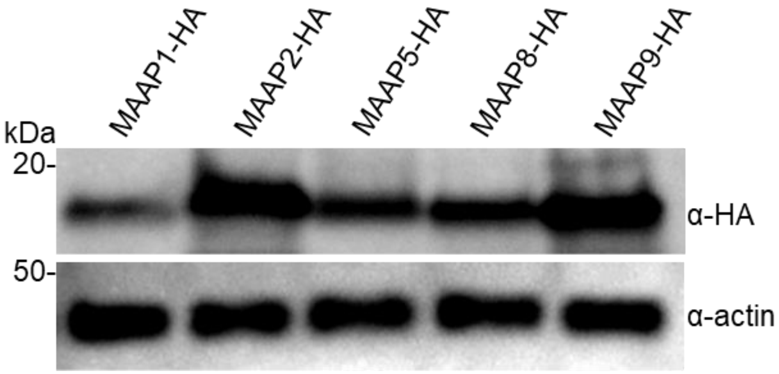

Supplementary Figure 5. Expression of MAAP-HA fusions. Anti-HA immunoblot of whole-cell extracts prepared from HEK293 cells expressing indicated HA tagged constructs. Anti-actin immunoblot served as loading control. Immunoblot is a representative image of 4 independent experiments.

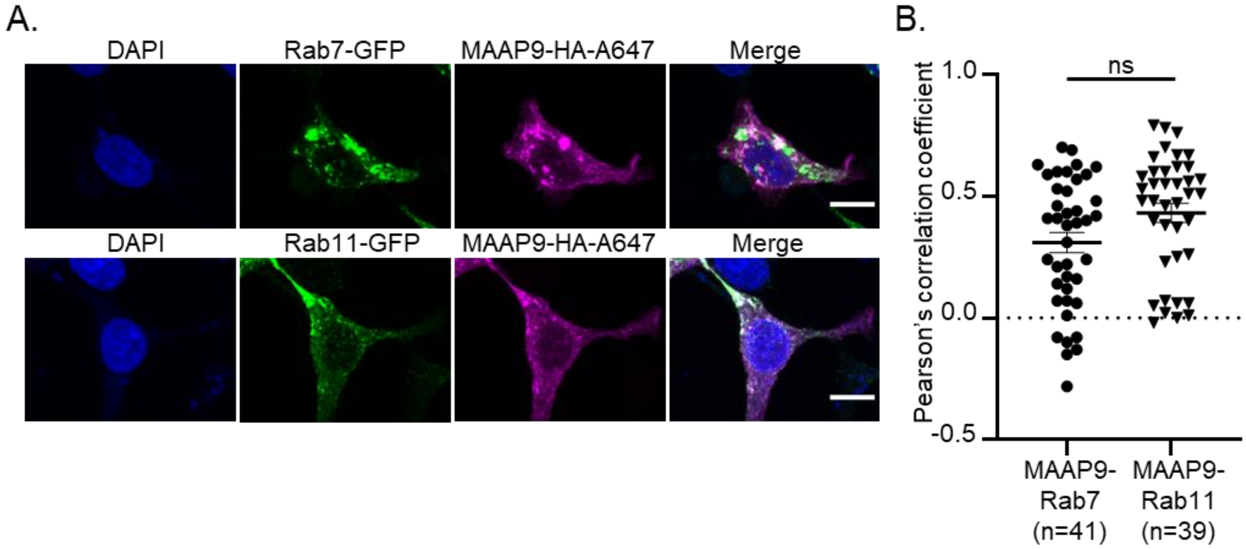

Supplementary Figure 6. MAAP9 demonstrates similar colocalization with endocytic and exocytic vesicular markers. (A) HEK293 cells were transfected with expression vectors encoding Rab7-GFP, Rab11-GFP, MAAP9-HA. MAAP-HA was detected by immunofluorescence with an AlexaFlour647 secondary antibody (MAAP9-HA-A647). A Z-stack of confocal optical sections at 1-µm steps was acquired. A 3-µm-thick medial stack is shown. Images are representative of three experiments. Scale bars, 10 µm. (**B**) Colocalization between MAAP9-HA and Rab7-GFP or Rab11-GFP in the whole cell as assessed by Pearson's correlation coefficient (R) as described in Materials and Methods. Each dot represents one cell. Horizontal bars represent the mean ± SEM, A two-sided Mann-Whitney rank test was used to determine significance. $^{n.s.}p \geq 0.05$

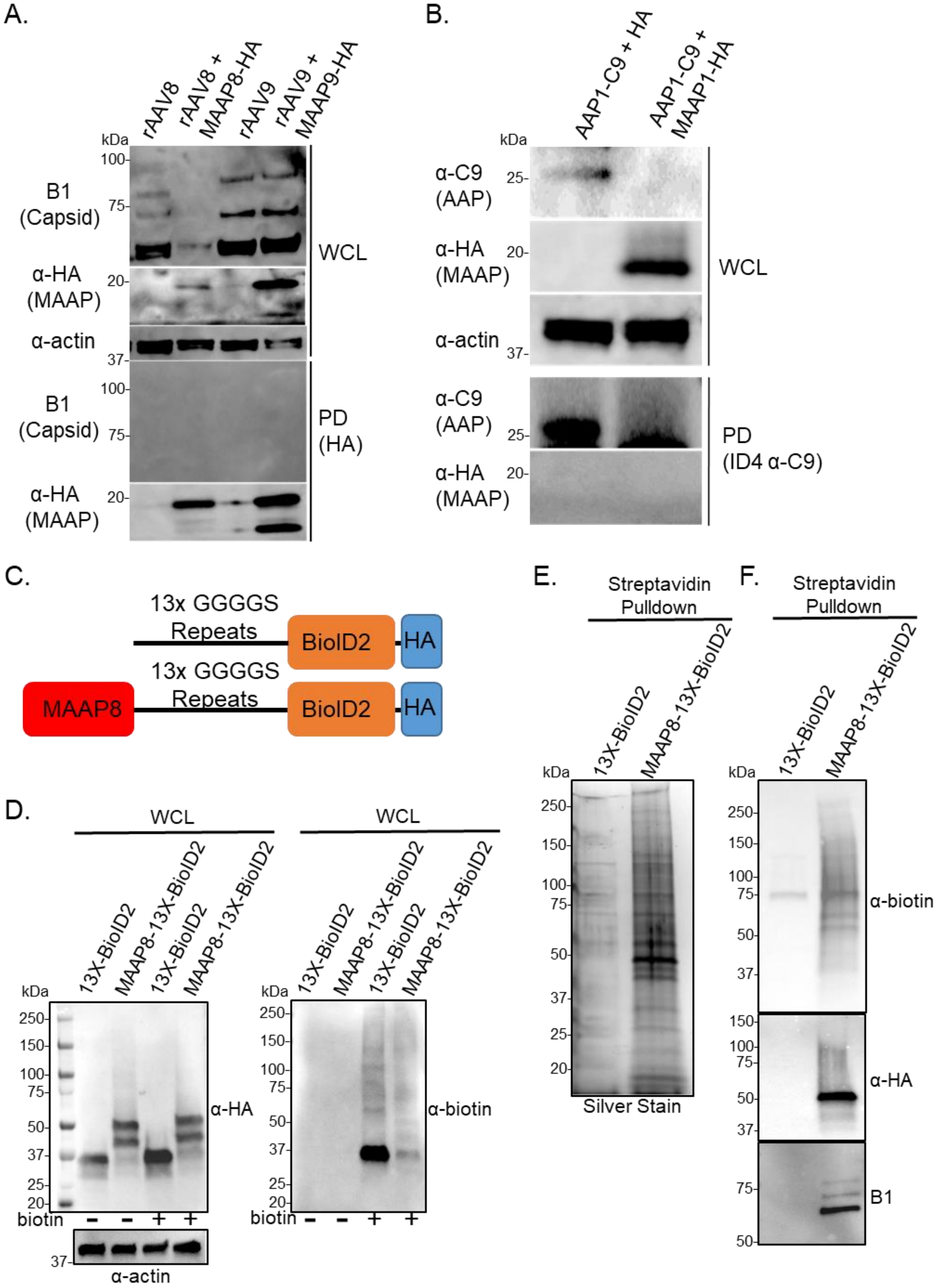
A.
rAAV8
rAAV8 + MAAP8-HA
rAAV9
rAAV9 + MAAP9-HA
kDa
B1 (Capsid)
α-HA (MAAP)
α-actin
WCL
PD (HA)
B.
AAP1-C9 + HA
AAP1-C9 + MAAP1-HA
α-C9 (AAP)
α-HA (MAAP)
α-actin
WCL
PD (ID4 α-C9)
C.
13x GGGGS Repeats
BioID2
HA
MAAP8
D.
WCL
13X-BioID2
MAAP8-13X-BioID2
α-HA
α-biotin
biotin
α-actin
E.
Streptavidin Pulldown
Silver Stain
F.
Streptavidin Pulldown
α-biotin
α-HA
B1

Supplementary Figure 7. MAAP proximally interacts with the AAV capsid.

(A) Immunoprecipitation (IP) of MAAP8/9-HA with rAAV8 and rAAV9 capsids and immunoblotting of input whole cell lysate (WCL) and pull down (PD) material for actin, capsid (B1), and MAAP8/9 (HA). **(B)** IP of AAP1-C9 and MAAP1-HA and immunoblotting of input whole cell lysate (WCL) and pull down (PD) material for actin, capsid (B1), MAAP8/9 (HA) and AAP (ID4). Immunoblots are representative of 3 independent experiments. **(C)** Schematic of MAAP8-13X-BioID2-HA fusions. **(D)** HEK293 cells were transfected with expression vectors encoding 13X-BioID2 and MAAP8-13X-BioID2. Media was supplemented with 50 μM biotin 24 hours post transfection and cells were harvested 24 hours post biotin supplementation. Whole cell lysate (WCL) was analyzed by SDS-PAGE under reducing conditions and probed with HA (α-HA), biotin (α-biotin) and actin (α-actin) specific antibodies. **(E)** HEK293 cells were transfected with plasmids encoding either 13X-BioID2 or MAAP8-13X-BioID2 along with pXX680, pTR-CBA-Luciferase, and AAV8-MAAPΔ. Media was supplemented with 50 μM biotin 48 hours post transfection and cells were harvested 20 hours post biotin supplementation. Biotinylated proteins pulled down on streptavidin resin were separated by SDS-PAGE and visualized by silver stain or **(F)** probed with biotin (α-biotin), (α-HA), and capsid (B1) specific antibodies. Immuoblots are representative images of 3 independent experiments.

**Supplementary Table 1**	
**Sequencing Primer**	**Sequence**
CMV-F (Eton)	CGCAAATGGGCGGTAGGCGTG
BGH-R (Eton)	TAGAAGGCACAGTCGAGG
PH116	CTGCCTGCGATCTGGTCAATGTGG
13xBioID2-F	TCGGATCCCATCTTTTTTCGGA
**Primer**	**Sequence**
pLH8-MAAP-3xFLAG SDM FWD	CACGACATCGACTACAAGGACGACGACGACAAGTAGAGCCATCACCCCAGCGTTCTCCAGACTC
pLH8-MAAP-3xFLAG SDM REV	GTCCTTGTAGTCACCGTCGTGGTCCTTGTAGTCCCGGTCTCTTCTTTCCAGGAGCCTCCTTAGC
AAV8-MAAPnull-SDM-F	AGGAGCCCCGAAGCCCAAAGC
AAV8-MAAPnull-SDM-R	GGTTTCAGCGCCCACCACTCG
MAAP2 null F	GAACAATAAATGATTTAAATCAGGTATGGCTG
MAAP2 null R	CTTAACAGGTTCCTCAACCAG
MAAP5 null F	GAACAATAAATGATTTAAATCAGGTATGGCTTTTGTTGATCAC
MAAP5 null R	CCTGAAAGACTGCCTTTCCG
MAAP8 delta 1-Fwd	CAAAAGCAGGACGACGGCTACAAGTACCTCGG
MAAP8 delta 1-Rev	CCGAGGTACTTGTAGCCGTCGTCCTGCTTTTG
MAAP8 delta 2-Fwd	CGTCCGCCGAGCCAGGAAGCACCAGA
MAAP8 delta 2-Rev	TCTGGTGCTTCCTGGCTCGGCGGACG
MAAP8 delta 3-Fwd	CGCAGCGGCCCAGGCGGGTGAC
MAAP8 delta 3-Rev	GTCACCCGCCTGGGCCGCTGCG
MAAP8 delta 4-Fwd	GTATCTTCTTGCAGACCACCCGCCTGCAGCTG
MAAP8 delta 4-Rev	CAGCTGCAGGCGGGTGGTCTGCAAGAAGATAC
MAAP8 delta 5-Fwd	GCTTCTTGGCCTTGCCCCCAAAAGACGTATCTTC
MAAP8 delta 5-Rev	GAAGATACGTCTTTTGGGGGCAAGGCCAAGAAGC
MAAP8 delta 6-Fwd	GGCCAAGAAGCGGGAGGAAGGCGCTAAG
MAAP8 delta 6-Rev	CTTAGCGCCTTCCTCCCGCTTCTTGGCC

MAAP8 delta 1-4-Fwd	GCAAAAGCAGGACGACGGTCTGCAAGAAGATACG
MAAP8 delta 1-4-Rev	CGTATCTTCTTGCAGACCGTCGTCCTGCTTTTGC
MAAP8 delta 1-3-Fwd	GGATTGTCACCCGCCTCGTCGTCCTGCTTTTG
MAAP8 delta 1-3-Rev	CAAAAGCAGGACGACGAGGCGGGTGACAATCC
MAAP8 delta 1-2-Fwd	AGCAGGACGACGCGGCGGACGCAG
MAAP8 delta 1-2-Rev	CTGCGTCCGCCGCGTCGTCCTGCT
MAAP8-deltaC-(delta74-119)-REV	TGGTCCTTGTAGTCACTCGGCGTCGGCG
MAAP8-deltaC-(delta74-119)-FWD	CGCCGACGCCGAGTGACTACAAGGACCA
MAAP8-deltaL-(delta24-73)-REV	GCAGACGCTCCTGAAAGCCAGGAAGCACCA
MAAP8-deltaL-(delta24-73)-FWD	TGGTGCTTCCTGGCTTTCAGGAGCGTCTGC
MAAP8-deltaN-(delta2-24)-REV	GGGCGCTGAAACCTGACAAGTACCTCGGAC
MAAP8-deltaN-(delta2-24)-FWD	GTCCGAGGTACTTGTCAGGTTTCAGCGCCC
MAAP8-deltaNL-(delta2-73)-REV	GGGCGCTGAAACCTGTTCAGGAGCGTCTGC
MAAP8-deltaNL-(delta2-73)-FWD	GCAGACGCTCCTGAACAGGTTTCAGCGCCC
mAAP8 W47S QC F	TCGAGCACGACAAGGCCTACGACCAGCAGCTGCAGGCG
mAAP8 W47S QC R	GTAGGCCTTGTCGTGCTCGAGGGCCGCTGCGTCC
13x BioID ATG Fix QC F	CGCCTCCACCGGATCCCATGAATTCCGAAAAAAGAT
13x BioID ATG Fix QC R	ATCTTTTTTCGGAATTCATGGGATCCGGTGGAGGCG
13x mAAP8 BioID F	CTAGCTAGCATGGAGCCCCGAAGC
13x mAAP8 BioID R	CCGGATTACCGGTACCGGTCTCTTCTTTC
**gblock**	**Sequence**
mAAP2-null	GAACAATAAATGATTTAAATCAGGTATGGCTGCCGATGGTTATCTTCCAGAT<br>TGGCTCGAGGACACTCTCTCTGAAGGAATAAGACAGTGGTGGAAGCTCAAA<br>CCAGGCCCACCACCACCAAAGCCCGCAGAGCGGCATAAGGACGACAGCA<br>GGGGTCTTGTGCTTCCAGGGTACAAGTACCTCGGACCCTTCAACGGACTC<br>GACAAGGGAGAGCCGGTCAACGAGGCAGACGCCGCGGCCCTCGAGCACG<br>ACAAAGCCTACGACCGGCAGCTCGACAGCGGAGACAACCCGTACCTGAAG

	TACAACCACGCCGACGCGGAGTTTCAGGAGCGCCTTAAAGAAGATACGTC<br>TTTTGGGGGCAACCTCGGACGAGCAGTCTTCCAGGCGAAAAAGAGGGTTC<br>TTGAACCTCTGGGCCTGGTTGAGGAACCTGTTAAG
mAAP9-null	ATATAAGTGAGCCCAAACGGGTGCGCGAGTCAGTTGCGCAGCCATCGACG<br>TCAGACGCGGAAGCTTCGATCAACTACGCAGACAGGTACCAAAACAAATGT<br>TCTCGTCACGTGGGCATGAATCTGATGCTGTTTCCCTGCAGACAATGCGAG<br>AGAATGAATCAGAATTCAAATATCTGCTTCACTCACGGACAGAAAGACTGTT<br>TAGAGTGCTTTCCCGTGTCAGAATCTCAACCCGTTTCTGTCGTCAAAAAGG<br>CGTATCAGAAACTGTGCTACATTCATCATATCATGGGAAAGGTGCCAGACG<br>CTTGCACTGCCTGCGATCTGGTCAATGTGGATTTGGATGACTGCATCTTTG<br>AACAATAAATGATTTAAATCAGGTATGGCTGCCGATGGTTATCTTCCAGATT<br>GGCTCGAGGACAACCTTAGTGAAGGAATTCGCGAGTGGTGGGCTTTGAAA<br>CCAGGAGCCCCTCAACCCAAGGCAAATCAACAACATCAAGACAACGCTCG<br>AGGTCTTGTGCTTCCGGGTTACAAATACCTTGGACCCGGCAACGGACTCGA<br>CAAGGGGGAGCCGGTGAACGCAGCAGACGCGGCGGCCCTCGAGCACGAC<br>AAGGCCTACGACCAGCAGCTGAAGGCCGGAGACAACCCGTACCTCAAGTA<br>CAACCACGCCGACGCCGAGTTCCAGGAGCGGCTGAAAGAAGATACGTCTT<br>TTGGGGGCAACCTCGGGCGAGCAGTCTTCCAGGCCAAAAAGAGGCTTCTA<br>GAACCTCTTGGTCTGGTTGAGGAAGCGGCTAAGACGGCTCCTGGAAAGAA<br>GAGGCCTGTAGAGCAGTCTCCTCAGGAACCGGACTCCTCCGCGGGTATTG<br>GCAAATCGGGTGCACAGCCCGCTAAAAAGAGACTCAATTTCGGTCAGACTG<br>GCGACACAGAGTCAGTCCCAGACCCTCAACCAATCGGAGAACCTCCCGCA<br>GCCCCCTCAGGTGTGGGATCTCTTACAATGGCTTCAGGTGGTGGCGCACC<br>AGTGGCAGACAATAACGAAGGTGCCGATGGAGTGGGTAGTTCCTCGGGAA<br>ATTGGCATTGCGATTCCCAATGGCTGGGGGACAGAGTCATCACCACCAGC<br>ACCCGAACCTGGGCCCTGCCCACCTACAACAATCACCTCTACAAGCAAATC<br>TCCAACAGCACATCTGGAGGATCTTCAAATGACAACGCCTACTTCGGCTAC<br>AGCACCCCCTGGGGGTATTTTGACTTCAACAGATTCCACTGCCACTTCTCA<br>CCACGTGACTGGCAGCGACTCATCAACAACAACTGGGGATTCCGGCCTAA<br>GCGACTCAACTTCAAGCTCTTCAACATTCAGGTCAAAGAGGTTACGGACAA<br>CAATGGAGTCAAGACCATCGCCAATAACCTTACCAGCACGGTCCAGGTCTT<br>CACGGACTCAGACTATCAGCTCCCGTACGTGCTCGGGTCGG
mAAP8-null	GATGGTTATCTTCCAGATTGGCTCGAGGACAACCTCTCTGAGGGCATTCGC<br>GAGTGGTGGGCGCTGAAACCAGGAGCCCCGAAGCCCAAAGCCAACCAGC<br>AAAAGCAGGACGACGGCCGGGGTCTGGTGCTTCCAGGCTACAAGTACCTC<br>GGACCCTTCAACGGACTCGACAAGGGGGAGCCCGTGAACGCGGCGGACG<br>CAGCGGCCCTCGAGCACGACAAGGCCTACGACCAGCAGCTGCAGGCGGG<br>TGACAATCCGTACCTGAGGTATAACCACGCCGACGCCGAGTTTCAGGAGC<br>GTCTGCAAGAAGATACGTCTTTTGGGGGCAACCTAGGGCGAGCAGTCTTC<br>CAGGCCAAGAAGCGGGTTCTCGAACCTCTCGGTCTGGTTGAGGAAGGCGC<br>TAAGACGGCTCCTGGAAAGAAGAGACCGGTAGAGCCATC
mAAP5-null	GAACAATAAATGATTTAAATCAGGTATGGCTTTTGTTGATCACCCTCCAGAT<br>TGGTTGGAAGAAGTTGGTGAAGGTCTTCGCGAGTTTTTGGGCCTTGAAGCG<br>GGCCCACCGAAACCAAAACCCAATCAGCAGCATCAAGATCAAGCCCGTGG<br>TCTTGTGCTGCCAGGTTATAACTATCTCGGACCCGGAAACGGTCTCGATCG<br>AGGAGAGCCTGTCAACAGGGCAGACGAGGTCGCGCGAGAGCACGACATC<br>TCGTACAACGAGCAGCTTGAGGCGGGAGACAACCCCTACCTGAAGTACAA<br>CCACGCGGACGCCGAGTTTCAGGAGAAGCTCGCCGACGACACATCCTTCG<br>GGGGAAACCTCGGAAAGGCAGTCTTTCAGG

mAAP1-null	GCTCGAGGACAACCTCTCTGAGGGCATTCGCGAGTGGTGGGACTTGAAAC<br>CAGGAGCCCCGAAGCCCAAAGCCAACCAGCAAAAGCAGGACGACGGCCG<br>GGGTCTGGTGCTTCCAGGCTACAAGTACCTCGGACCCTTCAACGGACTAG<br>ACAAGGGGGAGCCCGTCAACGCGGCGGACGCAGCGGCCCTCGAGCACGA<br>CAAGGCCTACGACCAGCAGCTCAAAGCGGGTGACAATCCGTACCTGAGGT<br>ATAACCACGCCGACGCCGAGTTTCAGGAGCGTCTGCAAGAAGATACGTCTT<br>TTGGGGGCAACCTCGGGCGAGCAGTCTTCCAGGCCAAGAAGCGGGTTCTC<br>GAACCTCTCGGTCTGGTTGAGGAAGGCGCTAAGACGGCTCCTGGAAAGAA<br>ACGTCCGGTAGAGCAGTCGCCACAAGAGCCAGACTCCTCCTCGGGCATCG<br>GCAAGACAGGCCAGCAGCCCGCTAAAAAGAGACTCAATTTTGGTCAGACT<br>GGCGACTCAGAGTCAGTCCCCGATCCACAACCTCTCGGAGAACCTCCAGC<br>AACCCCCGCTGCTGTGGGACCTACTACAATGGCTTCAGGCGGTGGCGCAC<br>CAATGGCAGACAATAACGAAGGCGCCGACGGAGTGGGTAATGCCTCAGGA<br>AATTGGCATTGCGATTCCACATGGCTGGGCGACAGAGTCATCACCACCAG<br>CACCCGCACCTGGGCCTTGCCCACCTACAATAACCACCTCTACAAGCAAAT<br>CTCCAGTGCTTCAACGGGGGCCAGCAACGACAACCACTACTTCGGCTACA<br>GCACCCCCTGGGGGTATTTTGATTTCAACAGATTCCACTGCCACTTTTCAC<br>CACGTGACTGGCAGCGACTCATCAACAACAATTGGGGATTCCG
AAV8 VP*	ATCAAAAGGGTGGAGCCAAGAAAAGACCCGCCCCCAGTGACGCAGATATA<br>AGTGAGCCCAAACGGGTGCGCGAGTCAGTTGCGCAGCCATCGACGTCAGA<br>CGCGGAAGCTTCGATCAACTACGCAGACAGGTACCAAAACAAATGTTCTCG<br>TCACGTGGGCATGAATCTGATGCTGTTTCCCTGCAGACAATGCGAGAGAAT<br>GAATCAGAATTCAAATATCTGCTTCACTCACGGACAGAAAGACTGTTTAGAG<br>TGCTTTCCCGTGTCAGAATCTCAACCCGTTTCTGTCGTCAAAAAGGCGTAT<br>CAGAAACTGTGCTACATTCATCATATCATGGGAAAGGTGCCAGACGCTTGC<br>ACTGCCTGCGATCTGGTCAATGTGGATTTGGATGACTGCATCTTTGAACAA<br>TAAATGATTTAAATCAGGTATAGCTGCCGATGGTTATCTTCCAGATTGGCTC<br>GAGGACAACCTCTCTGAGGGCATTCGCGAGTGGTGGGCGCTGAAACCTGG<br>AGCCCCGAAGCCCAAAGCCAACCAGCAAAAGCAGGACGACGGCCGGGGT<br>CTGGTGCTTCCTGGCTACAAGTACCTCGGACCCTTCAACGGACTCGACAAG<br>GGGGAGCCCGTCAACGCGGCGGACGCAGCGGCCCTCGAGCACGACAAG<br>GCCTACGACCAGCAGCTGCAGGCGGGTGACAATCCGTACCTGCGGTATAA<br>CCACGCCGACGCCGAGTTTCAGGAGCGTCTGCAAGAAGATACGTCTTTTG<br>GGGGCAACCTCGGGCGAGCAGTCTTCCAGGCCAAGAAGCGGGTTCTCGA<br>ACCTCTCGGTCTGGTTGAGGAAGGCGCTAAGGAGGCTCCTGGAAAGAAGA<br>GACCGGTAGAGCCATCACCCCAGCGTTCTCCAGACTCCTCTACGGGCATC<br>GGCAAGAAAGGCCAACAGCCCGCCAGAAAAAGACTCAATTTTGGTCAGAC<br>AGGCGACTCAGAGTCAGTTCCAGACCCTCAACCTCTCGGAGAACCTCCAG<br>CAGCGCCCTCTGGTGTGGGACCTAATACAATAGCTGCAGGCGGTGGCGCA<br>CCAATAGCAGACAATAACGAAGGCGCCGACGGAGTGGGTAGTTCCTCGGG<br>AAATTGGCATTGCGATTCCACATAGCTGGGCGACAGAGTCATCACCACCAG<br>CACCCGAACCTGGGCCCTGCCCACCTACAACAACCACCTCTACAAGCAAAT<br>CTCCAACGGGACATCGGGAGGAGCCACCAACGACAACACCTACTTCGGCT<br>ACAGCACCCCCTGGGGGTATTTTGACTTTAACAGATTCCACTGCCACTTTTC<br>ACCACGTGACTGGCAGCGACTCATCAACAACAACTGGGGATTCCGGCCCA<br>AGAGACTCAGCTTCAAGCTCTTCAACATCCAGGTCAAGGAGGTCACGCAGA<br>ATGAAGGCACCATGACCATAGCCAATAACCTGACCAGCACCATCCAGGTGT<br>TTACGGACTCGGAGTACCAGCTGCCGTACGTTCTCGAT

Supplementary Table 1. List of primers and DNA sequences used in this study.
